# Oropharyngeal Candidiasis: Fungal Invasion and Epithelial Cell Responses

**DOI:** 10.1371/journal.ppat.1006056

**Published:** 2017-01-12

**Authors:** Marc Swidergall, Scott G. Filler

**Affiliations:** 1 Division of Infectious Diseases, Los Angeles Biomedical Research Institute at Harbor-UCLA Medical Center, Torrance, California, United States of America; 2 David Geffen School of Medicine at UCLA, Los Angeles, California, United States of America; Geisel School of Medicine at Dartmouth, UNITED STATES

## Introduction

Oropharyngeal candidiasis (OPC) occurs in a diverse group of patients. Risk factors for OPC include the use of dentures, corticosteroid inhalers, cigarettes, broad-spectrum antibiotics, and immunosuppressive and chemotherapeutic agents. Patients with HIV, diabetes, and iatrogenic or autoimmune-induced dry mouth are also at substantial risk for OPC. This infection is caused primarily by *Candida albicans*, a ubiquitous polymorphic fungus that is part of the normal microbiota of the gastrointestinal and reproductive tracts of healthy individuals. In order to persistently colonize the oropharynx, *C*. *albicans* must adhere to the epithelial cell lining of the oral mucosa while avoiding being killed by host antimicrobial factors. OPC develops when local host defenses are weakened, permitting the fungus to invade and damage oral epithelial cells. The epithelial cells respond to fungal infection by secreting antimicrobial peptides that directly kill the fungus and by releasing pro-inflammatory cytokines that recruit neutrophils to the focus of infection, where they can kill *C*. *albicans* and limit the extent of epithelial cell damage [1,2]. In this Pearl, we summarize recent advances in our knowledge of the pathogenesis of OPC, focusing on fungal-epithelial interactions.

## *C*. *albicans* Invades, Damages, and Stimulates Oral Epithelial Cells

**Fig 1:**
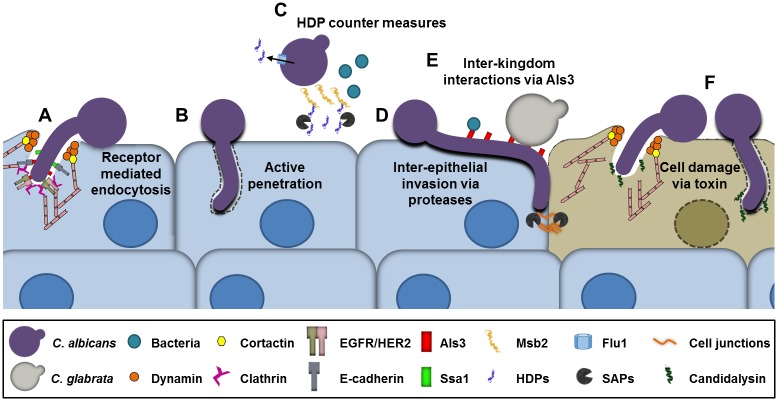
Diagram of the interactions of *Candida albicans* with oral epithelial cells, host defense peptides (HDPs), and the oral microbiota. (A) *C*. *albicans* invasion of epithelial cells by receptor-mediated endocytosis. The *C*. *albicans* Als3 and Ssa1 invasins interact with E-cadherin and a heterodimer composed of the epidermal growth factor receptor (EGFR) and HER2, which activate the clathrin endocytosis pathway, resulting in the endocytosis of the fungus. (B) *C*. *albicans* invasion by active penetration, in which a progressively elongating hyphus pushes its way into the epithelial cell. (C) Host defense peptides (HDPs) released by the infected epithelial cell can kill *C*. *albicans*. However, *C*. *albicans* can resist HDPs by up-regulating the Flu1 efflux pump, which reduces intracellular HDPs, by secreting aspartyl proteases (SAPs), which degrade HDPs, and by shedding of the Msb2 mucin, which binds to and inactivates HDPs. (D) *C*. *albicans* can invade between oral epithelial cells by proteolytic degradation of intercellular junctional proteins. (E) *C*. *albicans* hyphae bind *Candida glabrata* and bacteria such as *Staphylococcus aureus* and *Streptococcus* spp. *C*. *albicans* can enhance the capacity of some of these organisms to invade epithelial cells, while some of these organisms can increase the virulence of *C*. *albicans*. (F) *C*. *albicans* secretes candidalysin, a toxin that causes epithelial damage.

Although OPC is a superficial fungal infection, it is characterized by invasion of the epithelial cell lining of the oropharynx [3]. *C*. *albicans* can invade into oral epithelial cells by two distinct mechanisms, induced endocytosis and active penetration (Fig 1). During induced endocytosis, *C*. *albicans* hyphae express the Als3 [4] and Ssa1 [5] invasins, which bind to E-cadherin and a heterodimer composed of the epidermal growth factor receptor (EGFR) and HER2 on the epithelial cell surface [6] (Fig 1). These binding events trigger the clathrin-dependent endocytosis machinery and induce epithelial cells to produce pseudopods that engulf the fungus and pull it into the cell. Treatment of mice with a small molecule inhibitor of EGFR/HER2 reduces the severity of OPC, suggesting that induced endocytosis contributes to the pathogenesis of this infection [6]. Additional epithelial cell signaling pathways that are required for maximal endocytosis of *C*. *albicans* include the platelet-derived growth factor BB (PDGF BB) and neural precursor-cell-expressed developmentally down-regulated protein 9 (NEDD9) pathways. Activation of these signaling pathways requires *C*. *albicans* hyphal formation and expression of the Als3 invasin [7]. The relationship among these pathways and those activated by E-cadherin and EGFR/HER2 has not yet been determined.

Active penetration occurs when elongating *C*. *albicans* hyphae physically push their way into the epithelial cell (Fig 1) [8]. While it is likely that both induced endocytosis and active penetration occur during OPC, it has been difficult to determine the role of active penetration in the pathogenesis of OPC because most invasins are hyphal specific. Thus, *C*. *albicans* mutants that do not form hyphae also fail to express invasins and, consequently, are defective in both active penetration and induced endocytosis.

*C*. *albicans* can also penetrate epithelial cell barriers via a paracellular route by secreting lytic enzymes such as members of the secreted aspartyl proteinase (SAP) family. These proteases degrade E-cadherin and other inter-epithelial cell junctional proteins, enabling the organism to penetrate between epithelial cells [9] (Fig 1). Recently, it has been found that *C*. *albicans* infection also stimulates the activity of epithelial cell calpain, a cysteine protease that degrades E-cadherin. Moreover, calpain activity is dramatically enhanced in epithelial cells that are co-infected with *C*. *albicans* and *Streptococcus oralis* [10].

When *C*. *albicans* invades epithelial cells, it damages them. Epithelial cells in turn respond to *C*. *albicans* by secreting proinflammatory cytokines and antimicrobial peptides. Recently, it was discovered that *C*. *albicans*-induced epithelial cell damage is mainly caused by candidalysin, a toxin released by the fungus (Fig 1). Candidalysin is encoded by the hyphal-specific gene *ECE1*. The protein is cleaved by the Kex2 protease into eight peptides, of which only one permeabilizes epithelial cell membranes, induces epithelial cell damage, and stimulates epithelial cell cytokine secretion. A *C*. *albicans ece1*Δ/Δ deletion mutant is defective in damaging and stimulating oral epithelial cells in vitro. Furthermore, it has a greatly attenuated virulence in the mouse model of OPC [11]. Thus, candidalysin and epithelial cell damage play a central role in the pathogenesis of OPC.

Although the presence of candidalysin is required to induce a maximal epithelial cell response to *C*. *albicans*, binding of yeast, yeast lysates, and Als3 to epithelial cells is sufficient to induce some epithelial cell responses even in the absence of this toxin. For example, contact with *C*. *albicans* yeast cells, which do not secrete candidalysin, stimulates phosphorylation of the Akt serine/threonine kinase within 5 minutes [12]. In addition, lysates of yeast cells induce secretion of IL-8, possibly by interacting with intercellular adhesion molecule-1 (ICAM-1) [13]. Finally, *Saccharomyces cerevisiae* cells expressing the Als3 invasin stimulate the phosphorylation of EGFR and HER2 within 20 minutes [6]. These data suggest that oral epithelial cells sense and respond to contact with *C*. *albicans*, and that this initial response is subsequently amplified by candidalysin. While EGFR/HER2 and E-cadherin interact with *C*. *albicans* hyphae, the epithelial cell receptor(s) that is activated by yeast-phase *C*. *albicans* is currently unknown.

## Interplay between Epithelial Cell-Derived Antimicrobial Peptides and *C*. *albicans*

Host defense peptides (HDPs) represent one of the first lines of defense against invading microbes. Epithelial cells release multiple types of HDPs, including β-defensins and cathelicidins, that either kill or inhibit the growth of *C*. *albicans*. Different HDPs have different mechanisms of action, and their targets include the fungal cell membrane and mitochondria [14]. HDPs that are highly expressed during the early stage of OPC in mice include murine β-defensin 1 (mBD1), β-defensin 3 (the homolog of human β-defensin 2), and the alarmins S100A8 and S100A9 [15]. The importance of epithelial cell–derived HDPs in the host defense against OPC is demonstrated by finding that mBD1-deficient mice develop severe OPC, even in the absence of immunosuppression [16]. By contrast, immunocompetent wild-type mice are resistant to OPC, and when orally inoculated with *C*. *albicans*, clear the infection within 2–3 days. Mice that are deficient in either IL-17 receptor A (IL-17RA) or IL-17RC fail to up-regulate HDPs in the oral epithelium in response to *C*. *albicans* infection and are highly susceptible to OPC [1,15]. Recently, it was shown that mice with oral epithelial cell specific deletion of IL-17RA have reduced expression of *Defb3* (encoding β-defensin 3) in the oral mucosa and increased susceptibility to OPC. This increased susceptibility is phenocopied by deletion of *Defb3* [17]. Finally, people with mutations in signal transducer and activator of transcription 3 (STAT3) fail to mount a Th17 response, have significantly reduced salivary β-defensin 2 and histatins, and suffer from chronic mucocutaneous candidiasis [18]. Collectively, these data indicate that the response of oral epithelial cells to *C*. *albicans*, especially the production of HDPs, contributes to the host defense against OPC.

When *C*. *albicans* colonizes the oral mucosa of healthy individuals and causes OPC in patients, it must be able to withstand HDPs. The fungus has evolved multiple different mechanisms that enable it to evade the deleterious effects of HDPs and persist in the oral cavity [14]. These mechanisms include shedding of the Msb2 mucin, which binds to and inactivates HDPs, and secretion of aspartyl proteases, which break down HDPs [19,20,21] (Fig 1). *C*. *albicans* also expresses the Flu1 efflux pump that reduces intracellular HDP levels [22].

Exposure to HDPs induces a stress response in *C*. *albicans*, and susceptibility to HDPs is governed in part by the signaling pathways that are activated in response to these peptides. Activation of the high-osmolarity-glycerol (HOG) pathway enables *C*. *albicans* to resist histatin 5 and human β-defensins 2 and 3 [23,24]. The Ssd1 RNA-binding protein acts in part through the Bcr1 transcription factor to mediate resistance to human β-defensin 2 [25,26]. Conversely, activation of the Cek1 mitogen-activated protein kinase (MAPK) enhances surface exposure of β-1,3-glucans, elevating the uptake of histatin 5 and increasing sensitivity to this HDP [27].

## Co-infection with *C*. *albicans* and Other Microorganisms Influences Virulence during OPC

*C*. *albicans* is frequently isolated from the oral cavity in conjunction with other microbial pathogens. In a study of AIDS patients with OPC [28], 107 out of 1,106 episodes of infection (9.7%) were caused by more than one species of *Candida*. Among the subjects with multispecies OPC, *C*. *albicans* plus *C*. *glabrata* (68.2%) was the most common co-infection, followed by *C*. *albicans* plus *C*. *tropicalis* (15.0%) and *C*. *albicans* plus *C*. *glabrata* plus *C*. *tropicalis* (8.4%). These epidemiological data suggest that *C*. *glabrata* has unique characteristics that facilitate co-infection with *C*. *albicans*.

Silva et al. [29] reported that *C*. *glabrata* is unable to invade reconstituted human oral epithelium in vitro and causes minimal epithelial cell damage. A potential explanation for this finding is that *C*. *glabrata* grows only as yeast in vivo and does not form hyphae at foci of infection. However, when oral epithelial cells are infected with a mixture of *C*. *glabrata* and *C*. *albicans*, the *C*. *glabrata* cells bind to *C*. *albicans* hyphae and are carried deeper into the epithelium, where they cause extensive epithelial cell damage (Fig 1). It was recently reported that *C*. *glabrata* alone is not able to cause disease in a mouse model of OPC. However, when mice are inoculated with a mixture of *C*. *albicans* and *C*. *glabrata*, they develop more severe disease than mice infected with *C*. *albicans* alone [30]. This synergistic infection requires the expression of the Als1 and Als3 adhesins/invasins by the *C*. *albicans* hyphae. Multiple *C*. *glabrata* adhesins, including Epa8, Epa19, Awp2, Awp7, and CAGL0F00181, mediate adherence to *C*. *albicans* hyphae; whether they interact specifically with *C*. *albicans* Als1 and/or Als3 remains to be determined [30]. Collectively, these results provide an explanation for the frequent co-isolation of *C*. *glabrata* and *C*. *albicans* in patients with OPC. Although *C*. *tropicalis* can occasionally cause OPC in conjunction with *C*. *albicans*, the mechanistic basis for this co-infection is currently unknown.

Co-infection with *C*. *albicans* can also augment infection with *S*. *aureus*. When mice are immunosuppressed with cortisone acetate and then orally inoculated with *S*. *aureus*, the bacteria persist in the oral cavity in relatively low numbers and do not cause detectable disease [31]. However, when mice with OPC due to *C*. *albicans* are orally inoculated with *S*. *aureus*, the bacteria adhere to the fungal hyphae, which transport the bacteria along with them as they invade into the superficial oral epithelium. Not only does the presence of *C*. *albicans* result in higher levels of *S*. *aureus* in the oral cavity but it also enables *S*. *aureus* to invade the oral mucosa and cause a fatal hematogenously disseminated infection. This pathologic interaction between *S*. *aureus* and *C*. *albicans* is dependent on Als3; even though an *als3*Δ/Δ null mutant is still able to cause OPC, *S*. *aureus* co-infection does not result in a disseminated staphylococcal infection [31]. Although OPC is not known to be associated with disseminated staphylococcal infection in patients, it is possible that the interaction of *S*. *aureus* with *C*. *albicans* at other anatomic sites, such as the gastrointestinal tract or peritoneal cavity [32], might facilitate the development of *S*. *aureus* bacteremia.

*C*. *albicans* is also known to interact with common oral commensal bacteria. In vitro studies demonstrate that *Streptococcus gordonii* and *C*. *albicans* co-adhere during mixed-species biofilm formation [33]. This interaction is mediated by the *S*. *gordonii* cell wall-anchored proteins SspA and SspB and the *C*. *albicans* surface proteins Als3, Eap1, and Hwp1. The presence of *S*. *gordonii* enhances *C*. *albicans* hyphal development and increases biofilm mass [33,34,35]; whether it also augments *C*. *albicans* virulence during OPC is currently unknown. Co-infection with *Streptococcus mutans* also increases *C*. *albicans* biofilm formation in vitro. However, *Galleria mellonella* larvae infected with both organisms have prolonged survival compared to larvae infected with *C*. *albicans* alone. This reduction in virulence is likely due to inhibition of fungal hyphal formation by the bacteria [36]. By contrast, *Streptococcus oralis* both augments *C*. *albicans* biofilm formation in vitro and increases virulence during experimental OPC in mice, as manifested by increased size of the oral lesions and greater fungal dissemination to distal organs [37,38]. These data demonstrate that the interaction of *C*. *albicans* with the oral microbiota has the potential to significantly influence the pathogenesis of OPC.

## Conclusions and Perspective

The oral epithelium is a critical component of the host defense against OPC. However, *C*. *albicans* has evolved multiple strategies to breach the epithelial cell barrier, withstand HDPs, and cause a superficial infection. In mouse models of infection, other microbial pathogens can interact with *C*. *albicans* and alter virulence. This pathogenic interaction is undoubtedly relevant to human infection in the case of *C*. *glabrata* and streptococci because these organisms are commonly isolated in conjunction with *C*. *albicans*. Whether mucosal infection with *C*. *albicans* predisposes patients to develop invasive staphylococcal infections is currently unknown but is clearly worthy of investigation.
